# Characterization of New Egyptian Linseed Varieties and the Effects of Roasting on Their Pigments, Tocochromanols, Phytosterols, Omega-3 Fatty Acids, and Stability

**DOI:** 10.3390/molecules27238526

**Published:** 2022-12-03

**Authors:** Minar Mahmoud M. Hassanein, Adel Gabr Abdel-Razek, Sherine Mohamed Affifi, Ying Qian, Elżbieta Radziejewska-Kubzdela, Aleksander Siger, Magdalena Rudzińska, Ghada Ahmed Abo-Elwafa, Anna Grygier

**Affiliations:** 1 Department of Fats and Oils, National Research Center, Cairo 12622, Egypt; 2 Faculty of Food Science and Nutrition, Poznań University of Life Sciences, Wojska Polskiego 31, 60-624 Poznań, Poland

**Keywords:** linseed oil, roasting, pigments, tocochromanols, phytosterols, omega-3 fatty acids, stability

## Abstract

The purpose of this study was to explore the effects of roasting linseeds on the pigment, lipid profile, bioactive components, and oxidative stability of the extracted oils. The linseed varieties Giza 11, Giza 12, Sakha 3, and Sakha 6 were roasted at 180 °C for 10 min, and the oils were extracted by cold pressing. The results showed that, after roasting, there was an increase in oil percentage and peroxide value, as well as small increases in *p*-anisidine and acid values. Roasting also caused an increase in chlorophyll content, while lutein and β-carotene tend to slightly decrease, except in the Giza 11 variety. The total phenolics content was markedly enhanced after roasting. Omega-3 fatty acids were not affected by the roasting process. The total amounts of tocochromanol were found to decrease in the Giza 12 and Sakha 6 varieties after roasting. Plastochromanol-8 increased in all varieties after roasting. The phytosterol composition was minimally affected by roasting. Roasting enhanced the stability of the extracted oils, increasing the induction period and decreasing EC50 values. These results may thus help to discriminate between the different linseed varieties and serve to recommend the use of roasting to enhance the oxidative stability of extracted oil.

## 1. Introduction

Linseed (*Linum usitatissimum* L., also called flaxseed) is thought to be one of the oldest cultivated crops, with evidence of its cultivation dating back about five thousand years, when it was used for food and textile fiber purposes. The plant is now grown as both a fiber crop and an oil crop. Its global production was 3.18 million metric tons in 2018 [1]. 

Linseed oil is primarily used for industrial purposes, such as in the production of paints, linoleum floor coverings, and antirust agents, as well as being an additive to PVC plastic and an agglomerating agent for coal [2]. The crude linseed oil produced by cold pressing consists primarily of triacylglycerols but also contains smaller amounts of incomplete acylglycerols (monocylglycerols and diacylglycerols), sterols, tocopherols, phenolics, pigments (carotenoids and chlorophylls), and phospholipids [2]. These bioactive compounds make up the so-called unsaponifiable fraction, comprising about 0.39% to 0.78% of the total linseed oil [3,4]. The content and composition of this fraction plays an important role in retarding oil deterioration.

Linseeds contain 40–50% oil, 23–34% protein, 18% dietary fiber, and are rich in major and minor bioactive components. The oil extracted from linseeds is now becoming more important for its nutritional and pharmaceutical values, due to its high content of an omega-3 (ω-3) fatty acid, α-linolenic acid, which is one of the essential fatty acids that cannot be synthesized in the human body and must be obtained from food. Some studies have demonstrated that the intake of linseed oil can reduce many diseases, such as cancer and diabetes. These health benefits are associated with the presence of ω-3 fatty acid [2,5,6,7,8].

Roasting is a major step in making condiment oils, as it improves the color, flavor, composition, and quality of the extracted oils, which is important for consumer satisfaction [9,10]. Many studies have demonstrated that roasting can accelerate the Maillard reaction, enhance the flavor of oilseeds, and improve oil quality [11,12]. 

New varieties of linseeds were recently cultivated in Egypt, and no studies have studied their characterization. The objective of this work was thus to characterize the lipid profile of four new Egyptian linseed varieties and to evaluate how roasting affects the physicochemical properties, bioactive components (phytosterols, tocochromanols, TPC, pigments, fatty acids, triacylglycerol), and oxidative stability of the oil extracted from these varieties. 

## 2. Results and Discussion

### 2.1. Physicochemical Changes during Roasting 

#### 2.1.1. Proximate Chemical Analysis

We noted that the moisture content of the seeds decreased after roasting (Table 1). This is due to the fact that the roasting was carried out at a high temperature (180 °C), causing part of the water to evaporate and increasing the percentage of oil; however, in the Giza 11 sample the oil percentage remained nearly constant. The percentage of oil in sesame seeds increased during roasting [13], but that of oil in the wild almonds did not change [14].

Acid value (AV) is an important qualitative parameter; it is given in Table 1 for the unroasted and roasted forms of the varieties of linseed oils. The AVs of the unroasted Giza 11, Giza, 12, Sakha 3, and Sakha 6 seed oils were 0.86, 0.98, 0.63, and 0.68 mg KOH/g, respectively. As the seeds were roasted, the AVs slightly increased; this was in agreement with the results of Yoshida et al. [15], who found that the AV of sunflower seed oil increased with roasting. This increase in oil AV may be attributed to the hydrolysis of triacylglycerol (TAG) by heat to produce free fatty acids (FFA) and diacylglycerols (DAG), as reported by Yoshida et al. [16]. Fukuda [17] also reported that roasted sesame oil contained more FFAs than other unroasted seed oils. This slight increase in AV after roasting indicates that the roasted linseed oil is of good quality as, according to Codex Alimentarius [18], the AV did not exceed the limit of 4.0 mg KOH/g of oil.

The peroxide value (PV), which is based on the reduction of the hydroperoxide group with iodine ion (I-), provides information about the concentration of hydroperoxides. According to Table 1, peroxide values of the unroasted seeds of Giza 11 and 12 were higher than those of Sakha 3 and 6, so it is expected that, after roasting, the PV of Giza varieties will be higher than the Sakha varieties. However, it also could be noticed that the rate of increase in PV in all varieties was nearly similar. In general, all PV’s of all oil samples were less that the PV limit stated by Codex Alimentarius [18], which is 15 meq O2/kg oil. The PVs for all samples ranged from 2.90 to 6.72 meq O_2_/kg. According to lipid oxidation theory, hydroperoxides begin to accumulate at the initial stage of oxidation, reach a maximum, and then decrease as they decompose to secondary oxidation products. PVs thus increase with roasting in the initial stage of oxidation [19,20].

The *p*-Anisidine value (*p*-AV) is used to determine the quantity of aldehydes, and particularly that of 2-alkenals and 2,4-alkadienals. In almost all of the oil samples, the *p*-AVs remained nearly constant or slightly increased after roasting (Table 1). No change or a slight increase in the *p*-AV means that secondary oxidation products have not yet been formed.

TOTOX is often used to estimate the oxidative deterioration of lipids as it has the advantage of combining the amounts of primary oxidation products (hydroperoxides) with secondary products (principally alkenals and alkadienals) in fats or oils [21]. It can be noted from Table 1 that the TOTOX values of oils extracted from the roasted seeds were higher than those for the unroasted seeds—especially for the Giza varieties. The Sakha 3 variety showed the lowest TOTOX value (8.91) of all varieties (Table 1).

#### 2.1.2. Color Properties

The L* (lightness/darkness), a* (redness/greenness), and b* (yellowness/blueness) indices of unroasted and roasted seed oils are given in Table 2. The color values of the unroasted oil of the Giza varieties were 89.77, 1.35, and 59.34 for Giza 11 and 86.19, 5.47, and 57.15 for Giza 12. It was noticeable that the color darkened after roasting, with the values becoming 79.39, 4.07, and 52.54 for Giza 11 and 82.5, 6.59, and 54.71 for Giza 12, respectively. This may be due to the Millard reaction. The color values of the Sakha 3 variety increased after roasting. This may be due to the fact that Sakha 3 contains higher amounts of TPC after roasting (Table 2).

We analyzed the color using the photometric index at the wavelengths 460, 550, 620, and 670 nm, finding that the darkness increased with roasting for all flaxseed oil varieties other than the Giza 12 variety (7.23 before roasting, 4.08 after roasting), as shown in Table 2. This variety had the highest chlorophyll content (79.88 µg/kg) and the highest TPC (56.65 mg GAE/kg) after roasting. Totlani and Peterson [22] found that phenolic compounds can be used as inhibitors for Millard reaction products (MRPs).

The color index was measured after roasting (on sixteen wavelengths from 400 to 550 nm); it can be seen in Table 2 that the color values of the Giza 12 and Sakha 3 linseed oils decreased from 361.90 to 349.58 and from 290.78 to 274.57, respectively. This decrease may be due to the higher values of chlorophyll content and TPC (Table 2), as reported by Totlani and Peterson [22]. The darkness of oils after roasting reflects the formation of browning substances from nonenzymatic Millard-type reactions between reducing sugars and free amino acids or amides. Some derivatives of the Millard reaction also afford numerous food safety benefits [23].

#### 2.1.3. Chloroplast Pigments

Chlorophyll and lutein were the main chloroplast pigments present in the roasted and unroasted linseed oils (Table 3). In Giza 11 and Giza 12, chlorophyll increased from 23.13 and 47.25 in the oils of unroasted seeds to 38.63 and 79.88 µg/kg after roasting, respectively. In Sakha 3 and Sakha 6, it increased from 8.88 and 17.50 µg/kg in the oils of unroasted seeds to 11.25 and 26.75 µg/kg for the roasted seeds, respectively. We noted that the chlorophyll percentage increased by about 69.00%, 67.00%, 52.86%, and 26.69% in the linseed oils extracted from the roasted seeds of Giza 12, Giza 11, Sakha 6, and Sakha 3, respectively. This may be due to the breakdown of bonds between proteins and pigments, which increased the propagation of lipid-soluble pigments into the oil, thus improving oxidative stability [24].

β-Carotene, neoxanthin, violaxanthin, luteoxanthin, antheraxanthin, mutatoxanthin, and α-carotene were also identified alongside lutein (Table 3). Lutein is often reported to be the major carotenoid in linseed oil (roasted and unroasted), and there were also small amounts of β-carotene [25,26]. We noted that the content of lutein and β-carotene in linseed oil was extracted from the roasted seeds tended to decrease slightly after roasting. These results agree with those of Dumaz and Gokmen [27], who found that the levels of lutein and β-carotene in *Pistacia terebinthus* oil tend to decrease during roasting at 180 °C. Suresh et al. [28] reported that the decline in these compounds’ levels might be attributed to thermal degradation or polymerization when the oil is heated. It was only in the roasted Giza 11 oil that lutein and β-carotene increased with roasting (from 25.80 to 32.50 mg/kg and from 0.29 to 0.36 mg/kg, respectively). It could be noticed from Table 1 that Giza 11 is the only variety in which the oil content did not increase by roasting, while the oil contents of all other varieties were increased. The increase in oil content in Giza 12 and Sakha 3 and 6 could be explained by the dilutions to carotenoids pigments that appeared to decrease their content of lutein, β-carotene, and total carotenoids in general, in addition to the effect of roasting. These results agreed with those of Carrin and Carelli (2010) who reported that when an increase in oil content is accompanied by a reduction in carotenoid concentration, it is due to a dilution effect by the oil [29]. A slight increase in neoxanthin, luteoxanthin, mutatoxanthin, and α-carotene was recorded after roasting. Consequently, the total amount of carotenoids increased from 30 to 38 mg/kg oil. Chlorophyll and carotenoids may act as protectors, capturing the free radicals like the effect of alpha-tocopherol [30].

#### 2.1.4. Total Phenolic Content

Our analysis of the bioactive compounds and antioxidant capacity of the oils (Table 2) show that phenolic compounds were markedly enhanced after roasting, which is closely related to the increased antioxidant capacity. Additionally, Babiker et al. [31] showed an increase in oxidative properties in hemp oil during roasting. We found that TPC increased from 17.72 and 28.09 for unroasted seed oils to 31.52 and 56.65 mg GAE/kg for roasted seed oils in Giza 11 and Giza 12, respectively. The TPC in the oils of the Sakha 3 and Sakha 6 varieties increased with roasting from 33.09 and 11.28 for unroasted seed oil to 48.78 and 28.05 mg GAE/kg for roasted seed oil, respectively. We noted that the greatest value of TPC in oil after roasting was recorded for the Giza 12 variety followed by Sakha 3. Abdel-Razek et al. [30] reported that TPC plays an important role in protecting vegetable oils against autoxidation.

Nie et al. [32] reported that the presence of polyphenols in *Paeonia lactiflora* seed oil was primarily related to their antioxidant activities. In addition, MRPs enhanced the reducing power of the oil, leading to increased antioxidant capacity after roasting [33]. The reducing ketones and melanoidin in the MRPs could chelate metal ions and scavenge free radicals, thus blocking the chain reaction of free radicals [34].

### 2.2. Effects of Roasting on Major Lipid Components

#### 2.2.1. Fatty Acid Composition

Table 4 shows the fatty acid profiles of unroasted and roasted Egyptian linseed oils. Polyunsaturated fatty acids (PUFAs) were the major fatty acids in these profiles, with a predominance of α-linolenic acid (omega-3). The highest content of these components in the unroasted oil of the Giza 11 and Sakha 3 seeds amounted to 60.94% and 61.52%, respectively. The α-linolenic acid content was found to be 58.74% and 59.09% in the unroasted oil of Giza 12 and Sakha 6, respectively. The percentage of α-linolenic acid in Giza 11 and Sakha 3 is higher than that of German-originated oil linseed genotypes [35]. We note from the results shown in Table 4 that omega-3 was not noticeably changed by roasting. It was found that the Sakha 3 and Sakha 6 varieties contained greater amounts of omega-6 than the Giza varieties did (11, 12). Additionally, no marked change was noted in omega-6 after roasting; in fact, Giza 12 was found to contain greater amounts of omega-9 (20.12% and 20.05% for the unroasted and roasted oils, respectively) than the other three varieties and was also unaffected by roasting. The fatty acid profile of the oil extracted from the unroasted and roasted linseeds thus did not differ. 

The high stability of fatty acids during the heat treatment could be due to the relatively short roasting time of 10 min. Some studies have shown a slight effect on the fatty acid composition of linseed oil by roasting at 150–350 °C for 10 min [36] or at 160 °C for 8–24 min [37]. Other studies have indicated that there is no significant difference between the FA profiles of the oils of unroasted and roasted rapeseed and safflower seeds [38,39]. On the contrary, a significant effect of heating treatment on fatty acid profile was found in the case of sunflower and olive oils heated to 180 °C, 210 °C, and 240 °C for 15–120 min [40,41]. The difference between these results and our findings may indicate that the fatty acids of oils heated in a seed matrix (“in situ”) have higher stability than those heated outside this matrix.

The nutritional value of edible oils is determined by the MUFA:PUFA ratio, which remained nearly constant after roasting in all oil varieties. The omega-6:omega-3 ratio was found to be 0.21 and 0.18 in unroasted Giza 11 and Giza 12, respectively, and did not change after roasting. The same behavior was noted in Sakha 3 and Sakha 6, where the omega-6:omega-3 ratio amounted to 0.23 and 0.25 in unroasted oilseed to 0.22 and 0.25 in roasted seed oils, respectively. These values of the omega-6:omega-3 ratio before and after roasting are preferred as they can protect from some diseases, such as cardiovascular disease, cancer, and inflammatory and autoimmune diseases. Simpolous [42] reported that a lower omega-6:omega-3 fatty acid ratio is more desirable for reducing the risk of many of the chronic diseases that are highly prevalent in Western societies and developing countries, which are spreading to the rest of the world.

**Figure 1:**
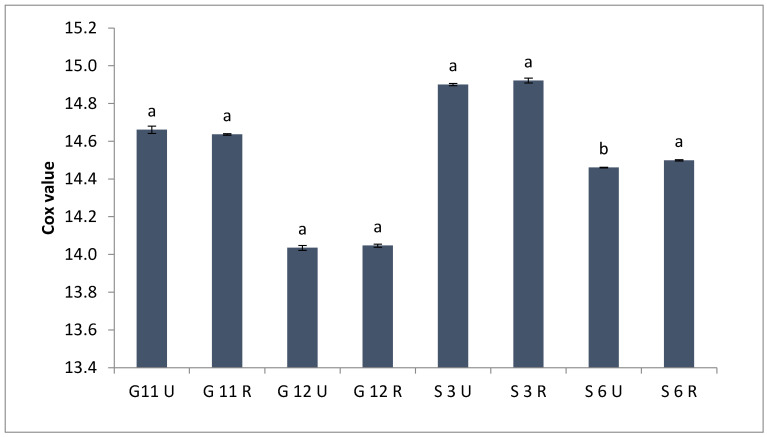
Cox values of oil extracted from four Egyptian unroasted and roasted linseed varieties. Values with different letters (^a^, ^b^) are significantly different (*p* < 0.05).

The cox value, calculated on the basis of the unsaturated fatty acids percentage of oils of unroasted and roasted linseeds, is presented in Figure 1. This value is commonly used as an indicator of an oil’s oxidation stability, with a lower value indicating a more stable oil [43,44]. It can be noted that roasting did not affect the cox value.

#### 2.2.2. Triacylglycerol

**Figure 2:**
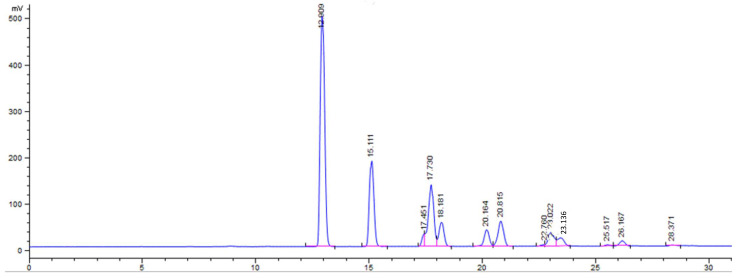
Chromatogram from triacylglycerol analysis.

Triacylglycerols (TAGs) are the main component of edible vegetable oils. Fourteen different TAGs were identified in our unroasted and roasted linseed oil (Table 5). Among the TAGs identified by HPLC, TAG LnLnLn (18:3/18:3/18:3) had the largest peak area, followed by TAG LnOLn (18:3/18:1/18:3) and TAG LnLLn (18:3/18:2/18:3). Other TAGs were found in reasonable amounts, such as LnLnP (18:3/18:3/16:0), LnLP (18:3/18:2/16:0), LLLn (18:2/18:2/18:3), and OOLn (18:1/18:1/18:3). The remaining TAGs were found in small amounts. A chromatogram of TAG analysis was shown in Figure 2.

The main TAG LnLnLn had the highest concentration of all the TAGs in all the varieties of linseed oil. It was found that LnLnLn was higher in unroasted seed oils and slightly decreased after roasting. In Giza 11, it decreased from 40.94% to 39.35%, while it remained nearly constant in Giza 12. The same behavior was observed in the other varieties, Sakha 3 and Sakha 6, which decreased after roasting from 44.17% and 39.30% to 42.64% and 37.26%, respectively. The second most common TAG was LnOLn in the Giza (11, 12) varieties and LnLLn in the Sakha (3, 6) varieties. It was noticeable that LnOLn increased after roasting in the Giza varieties but remained nearly constant in the Sakha varieties. Although the TAG LnLLn remained nearly constant after roasting in the Sakha varieties, it decreased after roasting in the Giza varieties. LnLnP, LnLP, and OOLn were found in reasonable amounts in all oil samples and almost did not change after roasting. Moreover, other TAGs found in small amounts were not significantly affected by roasting. Zhang et al. [12] reported that the main TAG in peanut oil was not remarkably different after roasting. Yoshida et al. [16] also illustrated that there was no clear difference in the fatty acid distribution of TAGs of peanuts during microwave roasting.

### 2.3. Effect of Roasting on Minor Lipid Components

#### 2.3.1. Tocochromanols

Tocochromanols are the most important natural antioxidants found in vegetable oils, though their antioxidant activity depends on their isomers and concentrations [45]. It has been shown that tocochromanols protect oils from oxidation and can prevent some diseases [46]. The results in Table 6 indicate that γ-tocopherol was the major isomer in the linseed oil samples, representing about 98% of their tocopherol content. We also found that both γ-tocopherol and total tocopherol contents of the oils from Giza 11 and Sakha 3 slightly increased after roasting, but noticeably decreased in the oils of the other two varieties (Giza 12 and Sakha 6) after roasting. Siger et al. [38] reported that roasting rapeseeds prior to pressing them resulted in a significant reduction in the levels of each tocopherol homolog.

Plastochromanol-8 (PC-8) was discovered fifty years ago in rubber tree leaves, where its content exceeded even that of α-tocopherol and plastoquinone. PC-8 occurs in the leaves of some plants, as well as in rape, maize, and linseed oils [47]. PC-8 is a third type of tocochromanol with a longer side chain, and it has antioxidant activities that can prevent photo-oxidative damage in leaves and oxidation processes in seed oils [47]. PC-8 noticeably increased in the linseed oils after roasting from 15.93 and 15.26 mg/100 g to 18.08 and 19.15 mg/100 g in Giza 11 and Giza 12, respectively, and from 15.77 and 14.74 mg/100 g to 23.13 and 17.62 mg/100 g in Sakha 3 and Sakha 6, respectively.

#### 2.3.2. Phytosterols

Table 6 shows the phytosterol profiles of the unroasted and roasted linseed oils. Sitosterol and Δ7-stigmasterol were the predominant phytosterols in the oil samples, followed by campesterol, Δ5-avenasterol, cycloartenol, stigmasterol, and 24-methylenecycloartanol. Campestanol and sitostanol were also detected. From the results in Table 6, it can be seen that a slight decrease in phytosterol composition in linseed oils of all varieties was noted after roasting, except in the case of Sakha 3, where a slight increase phytosterol abundance was recorded. On the other hand, when comparing the phytosterol profile of the unroasted linseed oils with the roasted oils, no noticeable differences were seen in the composition. 

In the current study, roasting may have caused only small changes in the phytosterol profiles of the roasted seed oil. On the other hand, the results in Table 6 may explain the stability of phytosterols under the roasting conditions used in our study as being due to short roasting time of 10 min at 180 °C. Zhang et al. [48] mentioned in their review that roasting contributes to an observed change in individual phytosterol contents but does not alter the composition of the phytosterols in the extracted oil.

### 2.4. Effects of Roasting on Oxidative Stability

#### 2.4.1. Antioxidant Activity

Antioxidant capacity reflects the presence of naturally existing and newly formed antioxidant constituents in extracted oil and is essential for assessing oil quality. Many methods are available to measure the antioxidant capacity of oil, including 1,1-diphenyl-2-picryl-hydrazylradical scavenging assays (DPPH), which are the most common and accessible of the methods [49]. The results in Table 7 indicate that EC50 decreased after the linseeds were roasted from 26.84, 25.97, 24.64, and 29.12 mg/mL to 24.52, 23.47, 23.01, and 26.18 mg/mL after roasting in Giza 11, Giza 12, Sakha 3, and Sakha 6, respectively. Różańska et al. [49] found that roasting improves the antioxidant activity.

Our analysis of the bioactive compound composition and antioxidant activity of linseed oils (Table 3 and Table 7) shows that the phenolic compounds were markedly enhanced by roasting; this is closely associated with the increased antioxidant activity. As we mentioned, the presence of polyphenols primarily related to their antioxidant activities and MRPs enhances the reducing power of the oil by chelating metal ions and scavenging free radicals, thus blocking the chain reaction of free radicals, leading to increased antioxidant activity after roasting [32,33,34].

On the other hand, we found that the RSA% increased in Giza 12 from 15.73% to 21.00% after roasting (Table 7), as this contains the greatest amount of phenolic compounds. The RSA% of the other varieties remained nearly constant after roasting.

#### 2.4.2. Induction Period

**Figure 3:**
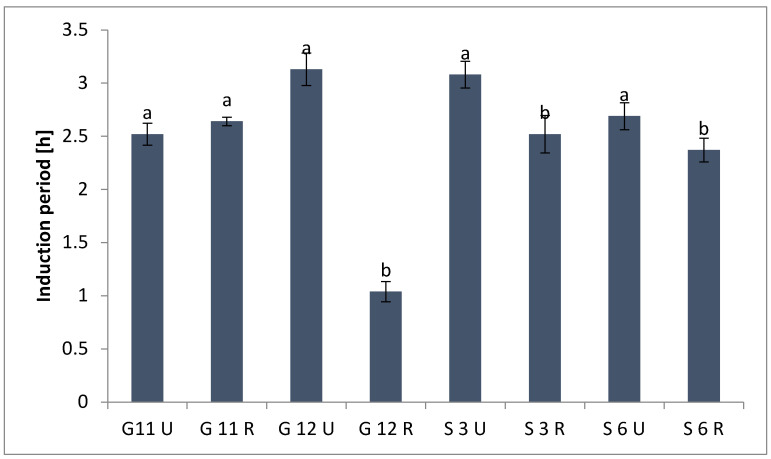
Induction period (h) of oil extracted from four Egyptian unroasted and roasted linseed varieties. Values with different letters (^a^, ^b^) are significantly different (*p* < 0.05).

The susceptibility of the studied roasted oils to oxidation was measured by the Rancimat test. The end point of the Rancimat test can be determined by the induction period (IP) to the inflection point in the oxidation curve [50]. The length of the IP is considered a relative measure of oil stability. As Figure 3 shows, there was a noticeable increase in IP after roasting in the Giza 12 and Sakha 3 varieties (3.08 h and 3.1 h, respectively), which may be due to the remarkable increase in phenolic content after roasting. In addition, a slight increase in IP in the case of roasted Giza 11 and a nearly constant value of IP in Sakha 6 were observed. 

The effect of roasting on the oxidative stability of oil can be attributed to two factors: One is that high temperature accelerates the oxidation of oil and destroys some heat-sensitive antioxidant compounds, leading to the generation of more oxidation products. The other is that roasting contributes to the better extraction of several natural antioxidants (such as tocopherols and polyphenols) by destroying the oilseed microstructure and generating MRPs with potential antioxidant activity, which results in improved oxidative stability of the oil. At present, MRPs prepared using reducing sugars, and enzymatically hydrolyzed protein of oilseeds are added into cold-pressed oil to improve its oxidation stability [51]. There is a balance between the formation of antioxidants and the degradation of endogenous antioxidants in roasted oilseeds [52]. Optimum roasting is thus necessary to maintain good oxidation stability of the oil. The oxidative stability of vegetable oils depends on many factors, such as tocochromanols, phytosterol contents, TPC, and fatty acid composition [53].

## 3. Materials and Methods

### 3.1. Plant Material

Four Egyptian linseed varieties (*Linum usitatissimum* L.)—namely, Giza 11, Giza 12, Sakha 3, and Sakha 6—were purchased from the Agricultural Research Centre, Giza, Egypt, in the 2021 season. The investigated linseed varieties were cultivated in north and south Delta, Egypt. The harvest time (maturation) was 145 days (between November to April). The seeds were divided equally into two portions: seeds that were left unroasted and seeds that were roasted and then sealed in paper bags and stored at 4 °C until use. 

### 3.2. Standards and Reagents

The 5α-cholestane, Folin–Ciocalteu reagent, phytosterol standards, carotenoids standards, and p-anisidine were purchased from Sigma-Aldrich (Munich, Germany). Tocochromanols (>95%, HPLC) were purchased from Merck (Darmstadt, Germany). The standard of fatty acid methyl esters mixture (FAME) was purchased from Supelco (Bellefonte, PA, USA). Other solvents and reagents were of analytical (ACS) or HPLC grade and were purchased from Sigma-Aldrich (Munich, Germany).

### 3.3. Roasting Treatment and Extraction of Oil from Linseeds

The roasting was conducted at 180 °C for ten minutes in a laboratory electric oven (1350 FX, SHEL LAB, Sheldon Manufacturing, Cornelius, NC, USA), where the seeds to be roasted were divided into three portions, each of about 300 g, and placed on a 15 × 30 cm metal tray in a thin layer form to ensure homogeneous thermal treatment. The three trays of each variety were placed in a pre-heated oven at the same time and roasted for ten minutes. After roasting, the seeds were cooled to room temperature and the divided portions were then mixed thoroughly and stored at 4 °C until extraction. 

The oil was extracted from the unroasted and roasted seeds of each variety at room-temperature using a stainless-steel screw press machine (Lewin, Henan, China). After centrifugation for 15 min at 5000 rpm, the oils were stored under nitrogen in closed, fully filled glass bottles in the dark at −20 °C until needed for analysis.

### 3.4. Determination of Seed Moisture and Oil Percentage

Seed moisture was determined using the oven method with a laboratory electric oven (1350 FX, SHEL LAB, Sheldon Manufacturing, Cornelius, NC, USA) at 105 °C, following the AOCS Official Method [54]. The oils were extracted with n-hexane from the varieties of linseeds (both roasted and unroasted) using the Soxhlet procedure for the determination of oil percentage, as described by the AOCS Official Method [55].

### 3.5. Physicochemical Characteristics of the Extracted Oils

#### 3.5.1. Oxidation Stability of Oils

Acid value (AV), peroxide value (PV), and p-anisidine value (p-AV) were determined in accordance with the AOCS Official Methods [56,57,58]. The total oxidation value (TOTOX) value was calculated using the formula: TOTOX = 2PV + p-AV.

#### 3.5.2. Determination of Color Properties

The color properties were determined using three different analysis methods:

- The color parameters under CIE Illuminant D65 and 1964 Standard Colorimetric Observer of oil samples were measured using a JASCO-V730 spectrophotometer (Jasco, Tokyo, Japan) with a 1.0 cm path-length quartz cell; the corresponding CIELAB coordinates were expressed as lightness (L), yellowness (b), and redness (a), in the cases of L (L = 0 = black, L = 100 = white), a (+a = red, −a = green), and b (+b = yellow, −b = blue) [59].
- Color measurements were carried out on a T80 UV/VIS spectrophotometer (PG Instruments, Lutterworth, United Kingdom) as follows:

- The photometric color index (PCI) was calculated using the following equation, based on the AOCS Official Method [60]:

PCI = 1.29(Ab460) + 69.7(Ab550) + 41.2(Ab620) − 56.4(Ab670),

where Ab460, Ab550, Ab620 and Ab670 are the absorbance values of oil at the wavelengths of 460 nm, 550 nm, 620 nm and 670 nm, respectively.

- The color index of the oil samples was calculated as the sum of sixteen absorbance values for the oil over the wavelength range of 400–550 nm (10 nm difference) multiplied by ten [61].

#### 3.5.3. Determination of Chloroplast Pigments

Chlorophyll was measured in accordance with the AOCS Official Method [62]. Briefly, dichloromethane was used as a comparative. The absorbance of oil samples was detected at 630 nm, 670 nm, and 710 nm, respectively. The concentration of the chlorophyll pigment was measured using the following equation:

$$C=\frac{A_{670}-\left(A_{630}+A_{710}\right)/2}{L}\times 1000,$$

where C is chlorophyll pigments (as µg/kg oil), A is an absorbance, and L is a cuvette length (4 cm).

Carotenoids were measured and identified following the method of Rodriguez-Amaya and Kimura [63] and Hart and Scott [64].

#### 3.5.4. Determination of Phenolic Compounds

Phenolic compounds were analyzed following Gutfinger et al. [65]; the method is described in detail in Naeem et al. [66].

### 3.6. Determination of Major Lipid Components

The major lipid components of the oil samples from both unroasted and roasted linseeds were analyzed.

#### 3.6.1. Fatty Acid Composition and Calculated Oxidizability Value (COX)

The fatty acid profile was analyzed using gas chromatography following the AOCS Official Method [67]. The calculated oxidizability value (COX) was calculated on the basis of unsaturated FA content using the following equation [44]:

COX = {1(18.1%) + 10.3(18.2%) + 21.6(18.3%)}/100,

where 18.1 is the percentage of oleic acid, 18.2 is the percentage of linoleic acid, and 18.3 is the percentage of linolenic acid.

#### 3.6.2. Triacylglycerol (TAG) Determinations

TAGs were determined on the basis of Ciftic et al. [68] and Idrus et al. [69]. For TAG analysis, HPLC with ELSD detector (1260 Infinity II, Agilent Technologies, Santa Clara, CA, USA) was used. The temperature of column (InfinityLab Poroshell 120 EC-C18 4.6 × 100 mm 2.7 Micron, Agilent Technologies, USA) was 30 °C. Initial composition of the mobile phase was phase A acetonitrile 80%, phase B dichloromethane 20%; up to 30 min, the phase change was 55% A and 45% B; then, up to 40 min, the composition of the phase was changed to the initial one and then held for 10 min. Samples were diluted in dichloromethane. ELSD parameters were as follows: evaporator and nebulizer temperatures were 30 °C, the gas flow rate was 1.60 SLM, the gain was (PM) 1.0.

### 3.7. Determination of Minor Lipid Components

Minor components were analyzed in the oil samples extracted from both unroasted and roasted linseeds.

#### 3.7.1. Tocochromanol Determinations

Tocochromanols were determined following Gawrysiak-Witulska et al. [70]. The unsaponifiable compounds were extracted using n-hexane/ethyl acetate (9:1 *v*/*v*). For tocochromanol determination, the samples were analyzed by HPLC. A LiChrosorb Si60 column (250 × 4.6 mm; 5 µm; Merck KGaA, Darmstadt, Germany) was used and the flow rate was 1.0 mL/min. The mobile phase consisted of n-hexane and 1,4-dioxane (96:4 *v*/*v*). The excitation wavelength of fluorometric detector was 295 nm and emission wavelength was 330 nm. 

#### 3.7.2. Phytosterol Profile

The phytosterol profile was determined according to the method described in detail in Hassanien et al. [71]. Firstly, samples were saponified with 1M KOH in methanol. Then, the unsaponifiables were extracted three times with hexane/methyl tert-butyl ether (1:1, *v*/*v*). The solvents were evaporated and derivatization were made. The samples were analyzed by GC-FID HP 6890 equipped with a DB-35MS capillary column (25 m × 0.20 mm; 0.33 μm; J&W Scientific, Folsom, CA, USA). At the beginning of the analysis, the column temperature was 100 °C for 5 min, then programmed to rise to 250 °C at 25 °C min^−1^, held for 1 min, and further programmed to rise to 290 °C at 3 °C min^−1^, where it was held for 20 min.

### 3.8. Determination of Oxidative Stability Parameters

#### 3.8.1. DPPH Free Radical Scavenging Assay

Radical scavenging activity (RSA%) and EC 50 (the minimum effective concentration of extract required to scavenge DPPH radical by 50%) was determined according to Ramadan et al. [72].

#### 3.8.2. Rancimat Test

The induction period of the oil samples was determined following Kowalski et al.’s work [73], using a Metrohm Rancimat apparatus model 892 (Metrohm, Herisau, Switzerland) at 110 °C ± 0.1 °C and an air flow of 20 L/h to determine the induction period (IP).

### 3.9. Statistical Analysis

The results are presented as means ± standard deviations (SD) from three replicates of each experiment. The statistical program Statistica 13.3 (TIBCO Software Inc., Palo Alto, CA, USA) was used to prepare one-way analysis of variance (ANOVA). The Tukey test was used, with a significance level of α = 0.05. The analyses were performed comparing roasted and unroasted oil of the same variety.

## 4. Conclusions

The four new Egyptian linseed varieties showed the high concentrations of omega-3 fatty acids (18:3), ranging from 58.74% to 61.52%. γ-Tocopherol was the major isomer in the linseed oil samples, representing about 98% of the tocopherol content. Roasting the Egyptian linseed varieties at 180 °C for 10 min enhanced the physicochemical properties of their extracted oils. It was found that PC-8 noticeably increased in linseed oils after roasting. Additionally, the content of certain bioactively important components, such as chlorophyll, TPC, and tocochromanols, also increased, enhancing the oxidative stability of the oil, as shown by the increased value of IP and the decreased EC50. Neither omega-3 nor omega-6 oils noticeably changed after roasting. Omega-6 was found to be higher in the Sakha 3 and Sakha 6 varieties than in Giza 11 and Giza 12. However, Giza 12 was found to contain greater amounts of omega-9. The oxidative stability of linseed oils depends on many factors, such as tocochromanols, phytosterol contents, TPC, and fatty acid composition.

## Figures and Tables

**Table 1:** Physicochemical characteristics of oils extracted from four Egyptian unroasted and roasted linseed varieties.

	Egyptian Linseed Cultivars							
Parameters	Giza 11		Giza 12		Sakha 3		Sakha 6	
	U	R	U	R	U	R	U	R
**Moisture content (%)**	6.94 ^a^ ± 0.02	2.62 ^b^ ± 0.11	6.38 ^a^ ± 0.20	2.12 ^b^ ± 0.01	6.26 ^a^ ± 0.02	3.02 ^b^ ± 0.10	6.89 ^a^ ± 0.05	3.42 ^b^ ± 0.11
**Oil percentage (%)**	38.20 ^a^ ± 0.20	38.03 ^a^ ± 0.29	36.35 ^b^ ± 0.15	38.88 ^a^ ± 0.40	39.05 ^b^ ± 0.31	40.78 ^a^ ± 0.46	41.39 ^a^ ± 0.02	42.01 ^a^ ± 0.57
**Acid value (mg KOH/g)**	0.86 ^a^ ± 0.04	0.88 ^a^ ± 0.04	0.98 ^b^ ± 0.00	1.15 ^a^ ± 0.04	0.63 ^b^ ± 0.00	0.85 ^a^ ± 0.01	0.68 ^a^ ± 0.00	0.76 ^a^ ± 0.00
**Peroxide value (meq O_2_/kg)**	4.31 ^b^ ± 0.30	6.11 ^a^ ± 0.87	4.65 ^b^ ± 0.00	6.72 ^a^ ± 0.89	2.91 ^a^ ± 0.01	3.20 ^a^ ± 0.29	2.90 ^b^ ± 0.02	5.53 ^a^ ± 0.28
***p*** **-Anisidine value** **(mmol/kg)**	2.75 ^b^ ± 0.12	3.19 ^a^ ± 0.04	2.25 ^a^ ± 0.40	2.42 ^a^ ± 0.17	2.46 ^a^ ± 0.44	2.52 ^a^ ± 0.03	1.51 ^b^ ± 0.09	1.84 ^a^ ± 0.27
**Totox value**	11.36 ^b^ ± 0.71	15.41 ^a^ ± 1.70	11.55 ^b^ ± 0.40	15.85 ^a^ ± 1.94	8.27 ^b^ ± 0.43	8.91 ^a^ ± 0.56	7.31 ^b^ ± 0.13	12.89 ^a^ ± 0.38

Where: U = Unroasted, R = Roasted; Mean ± standard deviation; Number of replicates for each analysis: 3; Values with different letters (^a^, ^b^) are significantly different (*p* < 0.05).

**Table 2:** Analysis of color by various methods of oil extracted from four Egyptian unroasted and roasted linseed varieties.

	Egyptian Linseed Cultivars							
Color Methods	Giza 11		Giza 12		Sakha 3		Sakha 6	
	U	R	U	R	U	R	U	R
Color coordinates								
**L**	89.77 ^a^ ± 0.06	79.39 ^b^ ± 0.13	86.19 ^a^ ± 0.05	82.50 ^b^ ± 0.08	81.52 ^b^ ± 0.19	86.21 ^a^ ± 0.18	92.83 ^a^ ± 0.02	89.93 ^b^ ± 0.17
**a**	1.35 ^b^ ± 0.03	4.07 ^a^ ± 0.03	5.47 ^b^ ± 0.01	6.59 ^a^ ± 0.03	−1.03 ^a^ ± 0.04	−1.35 ^a^ ± 0.09	−3.75 ^b^ ± 0.01	−1.96 ^a^ ± 0.05
**b**	59.34 ^a^ ± 0.03	52.54 ^b^ ± 0.09	57.15 ^a^ ± 0.04	54.71 ^b^ ± 0.05	53.53 ^b^ ± 0.13	56.55 ^a^ ± 0.12	60.95 ^a^ ± 0.01	59.09 ^b^ ± 0.11
**Delta EH**	60.23 ^a^ ± 0.03	56.58 ^b^ ± 0.04	59.06 ^a^ ± 0.02	57.82 ^b^ ± 0.02	56.64 ^b^ ± 0.06	58.22 ^a^ ± 0.07	61.48 ^a^ ± 0.01	59.98 ^b^ ± 0.08
**Delta L**	−10.23 ^a^ ± 0.06	-20.61 ^b^ ± 0.13	−13.81 ^a^ ± 0.05	−17.50 ^b^ ± 0.08	−18.48 ^b^ ± 0.19	−13.79 ^a^ ± 0.18	−7.17 ^a^ ± 0.02	−10.07 ^b^ ± 0.17
**Delta a**	1.36 ^b^ ± 0.03	4.08 ^a^ ± 0.02	5.47 ^b^ ± 0.01	6.59 ^a^ ± 0.03	−1.02 ^a^ ± 0.04	−1.35 ^a^ ± 0.08	−3.75 ^b^ ± 0.01	−1.95 ^a^ ± 0.05
**Delta b**	59.34 ^a^ ± 0.03	52.54 ^b^ ± 0.09	57.16 ^a^ ± 0.04	54.71 ^b^ ± 0.05	53.53 ^b^ ± 0.13	56.55 ^a^ ± 0.12	60.95 ^a^ ± 0.01	59.09 ^b^ ± 0.11
**Photometric color**	8.82 ^b^ ± 0.89	10.89 ^a^ ± 0.82	7.23 ^a^ ± 1.93	4.08 ^b^ ± 0.86	10.43 ^b^ ± 0.84	11.82 ^a^ ± 1.40	7.26 ^b^ ± 0.49	9.45 ^a^ ± 0.43
**Color index**	342.41 ^b^ ± 0.56	353.72 ^a^ ± 3.18	361.90 ^a^ ± 2.93	349.58 ^b^ ± 4.28	290.78 ^a^ ± 2.41	274.57 ^b^ ± 2.27	272.35 ^b^ ± 0.32	288.89 ^a^ ± 1.08
**Total phenolic compounds (mg GAE/kg)**	17.72 ^b^ ± 0.65	31.52 ^a^ ± 3.89	28.09 ^b^ ± 9.35	56.65 ^a^ ± 4.94	33.09 ^b^ ± 1.87	48.78 ^a^ ± 4.21	11.28 ^b^ ± 1.21	28.05 ^a^ ± 6.01

Where: U = Unroasted, R = Roasted; L (lightness); a (negative values indicate green, positive values indicate red; −a/+a); b (negative values indicate blue, positive values indicate yellow; +b/−b); Mean ± standard deviation; Number of replicates for each analysis: 3; Values with different letters (^a^, ^b^) are significantly different (*p* < 0.05).

**Table 3:** Chloroplast pigments (chlorophyll and carotenoids) and total phenolic contents of oil extracted from four Egyptian unroasted and roasted linseed varieties.

	Egyptian Linseed Cultivars							
Chloroplast Pigments	Giza 11		Giza 12		Sakha 3		Sakha 6	
	U	R	U	R	U	R	U	R
**Chlorophyll content** **(µg/kg)**	23.13 ^b^ ± 0.53	38.63 ^a^ ± 0.38	47.25 ^b^ ± 0.60	79.88 ^a^ ± 0.38	8.88 ^b^ ± 0.73	11.25 ^a^ ± 0.75	17.50 ^b^ ± 0.50	26.75 ^a^ ± 1.05
**Carotenoids content (mg/kg oil)**								
**neoxanthin**	1.55 ^b^ ± 0.02	2.18 ^a^ ± 0.25	3.13 ^a^ ± 0.35	2.47 ^b^ ± 0.39	1.05 ^a^ ± 0.13	0.94 ^a^ ± 0.01	0.99 ^a^ ± 0.19	0.92 ^a^ ± 0.14
**violaxanthin**	0.56 ^a^ ± 0.01	0.51 ^a^ ± 0.05	1.14 ^a^ ± 0.12	0.38 ^b^ ± 0.04	—	—	—	—
**luteoxanthin**	0.59 ^b^ ± 0.01	0.81 ^a^ ± 0.10	1.43 ^a^ ± 0.14	1.12 ^b^ ± 0.16	0.43 ^a^ ± 0.04	0.27 ^b^ ± 0.01	0.29 ^a^ ± 0.00	0.25 ^a^ ± 0.04
**antheraxanthin**	0.48 ^a^ ± 0.00	0.44 ^a^ ± 0.01	0.51 ^a^ ± 0.07	0.24 ^b^ ± 0.03	0.40 ^a^ ± 0.05	0.27 ^a^ ± 0.06	0.35 ^a^ ± 0.07	0.25 ^a^ ± 0.00
**mutatoxanthin**	0.75 ^b^ ± 0.01	1.18 ^a^ ± 0.11	1.16 ^a^ ± 0.15	1.19 ^a^ ± 0.33	0.76 ^a^ ± 0.14	0.55 ^b^ ± 0.11	0.75 ^a^ ± 0.27	0.70 ^a^ ± 0.08
**lutein**	25.8 ^b^ ± 0.7	32.5 ^a^ ± 2.5	42.5 ^a^ ± 4.6	36.1 ^b^ ± 6.7	17.6 ^a^ ± 1.9	13.4 ^b^ ± 1.1	19.1 ^a^ ± 2.8	16.7 ^b^ ± 0.8
**α-carotene**	0.16 ^a^ ± 0.05	0.17 ^a^ ± 0.03	0.12 ^a^ ± 0.04	0.08 ^a^ ± 0.01	0.07 ^a^ ± 0.01	0.05 ^a^ ± 0.01	0.03 ^a^ ± 0.00	0.06 ^a^ ± 0.01
**β-carotene**	0.29 ^a^ ± 0.06	0.36 ^a^ ± 0.03	0.43 ^a^ ± 0.04	0.31 ^a^ ± 0.06	0.18 ^a^ ± 0.03	0.15 ^a^ ± 0.03	0.18 ^a^ ± 0.04	0.16 ^a^ ± 0.01
**total**	30 ^b^ ± 1	38 ^a^ ± 1	50 ^a^ ± 5	42 ^b^ ± 8	20 ^a^ ± 2	16 ^a^ ± 1	22 ^a^ ± 4	19 ^a^ ± 1

Where: U = Unroasted, R = Roasted; Mean ± standard deviation; Number of replicates for each analysis: 3; Values with different letters (^a^, ^b^) are significantly different (*p* < 0.05).

**Table 4:** Fatty acid composition of oil extracted from four Egyptian unroasted and roasted linseed varieties (%).

	Egyptian Linseed Cultivars							
Fatty Acids	Giza 11		Giza 12		Sakha 3		Sakha 6	
	U	R	U	R	U	R	U	R
**C 16:0**	5.01 ^b^ ± 0.02	5.17 ^a^ ± 0.00	5.23 ^b^ ± 0.01	5.32 ^a^ ± 0.05	5.13 ^a^ ± 0.01	5.17 ^a^ ± 0.02	5.16 ^b^ ± 0.03	5.24 ^a^ ± 0.00
**C 18:0**	4.52 ^a^ ± 0.01	4.40 ^b^ ± 0.03	4.78 ^a^ ± 0.09	4.72 ^a^ ± 0.02	4.15 ^a^ ± 0.01	4.12 ^a^ ± 0.00	4.84 ^a^ ± 0.03	4.57 ^b^ ± 0.01
**C 18:1 (w9)**	16.63 ^b^ ± 0.10	16.76 ^a^ ± 0.04	20.12 ^a^ ± 0.01	20.05 ^a^ ± 0.00	14.97 ^a^ ± 0.02	14.91 ^a^ ± 0.06	16.00 ^a^ ± 0.02	15.96 ^a^ ± 0.01
**C 18:2 (w6)**	12.87 ^a^ ± 0.01	12.73 ^b^ ± 0.10	11.14 ^a^ ± 0.04	11.10 ^a^ ± 0.01	14.21 ^a^ ± 0.00	14.13 ^a^ ± 0.02	14.92 ^a^ ± 0.07	14.97 ^a^ ± 0.01
**C 18:3 (w3)**	60.94 ^a^ ± 0.11	60.92 ^a^ ± 0.04	58.74 ^a^ ± 0.04	58.81 ^a^ ± 0.04	61.52 ^a^ ± 0.03	61.65 ^a^ ± 0.06	59.09 ^b^ ± 0.03	59.26 ^a^ ± 0.04
**MUFA/PUFA**	0.23 ^a^	0.23 ^a^	0.29 ^a^	0.29 ^a^	0.20 ^a^	0.20 ^a^	0.22 ^a^	0.22 ^a^
**n-6/n3**	0.21 ^a^	0.21 ^a^	0.19 ^a^	0.19 ^a^	0.23 ^a^	0.23 ^a^	0.25 ^a^	0.25 ^a^

Where: U = Unroasted, R = Roasted; Mean ± standard deviation; Number of replicates for each analysis: 3; Values with different letters (^a^, ^b^) are significantly different (*p* < 0.05).

**Table 5:** Abundance of triacylglycerols (%) in oil extracted from four Egyptian unroasted and roasted linseed varieties.

	Egyptian Linseed Cultivars							
Triacylglycerol	Giza 11		Giza 12		Sakha 3		Sakha 6	
	U	R	U	R	U	R	U	R
**LnLnLn**	40.94 ^a^ ± 0.89	39.35 ^b^ ± 0.54	37.32 ^a^ ± 0.18	37.65 ^a^ ± 0.42	44.17 ^a^ ± 0.39	42.64 ^b^ ± 0.39	39.30 ^a^ ± 0.46	37.26 ^b^ ± 1.36
**LnLLn**	15.65 ^a^ ± 0.09	14.03 ^b^ ± 1.66	14.17 ^a^ ± 1.60	12.22 ^b^ ± 0.01	16.87 ^b^ ± 0.10	17.23 ^a^ ± 0.04	17.83 ^a^ ± 0.03	17.56 ^a^ ± 0.09
**LLLn**	1.76 ^a^ ± 0.24	1.22 ^b^ ± 0.23	1.50 ^a^ ± 0.31	0.99 ^b^ ± 0.16	1.83 ^b^ ± 0.29	2.00 ^a^ ± 0.10	2.30 ^a^ ± 0.30	2.21 ^a^ ± 0.39
**LnOLn**	16.01 ^b^ ± 0.43	18.13 ^a^ ± 0.94	18.12 ^b^ ± 1.34	19.67 ^a^ ± 0.08	14.25 ^a^ ± 0.09	14.38 ^a^ ± 0.15	14.97^a^ ± 0.04	15.15 ^a^ ± 0.21
**LnLnP**	5.44 ^a^ ± 0.10	5.44^a^ ± 0.16	5.75 ^a^ ± 0.39	5.49 ^b^ ± 0.02	5.24 ^a^ ± 0.04	5.49 ^a^ ± 0.19	5.18 ^b^ ± 0.08	5.76^a^ ± 0.27
**OLLn**	3.74 ^a^ ± 0.12	3.72 ^a^ ± 0.12	3.90 ^a^ ± 0.37	3.60 ^b^ ± 0.05	3.66 ^a^ ± 0.01	3.77 ^a^ ± 0.01	4.46 ^a^ ± 0.19	4.65 ^a^ ± 0.33
**LnLP**	6.29 ^a^ ± 0.16	6.07 ^a^ ± 0.18	6.50 ^a^ ± 0.40	5.83 ^b^ ± 0.11	6.13 ^a^ ± 0.07	6.17 ^a^ ± 0.02	6.62 ^a^ ± 0.20	6.78 ^a^ ± 0.19
**OLO**	0.22 ^a^ ± 0.07	0.26 ^a^ ± 0.12	0.24 ^b^ ± 0.01	0.33 ^a^ ± 0.03	0.28 ^a^ ± 0.03	0.25 ^a^ ± 0.03	0.28 ^a^ ± 0.07	0.35 ^a^ ± 0.07
**OOLn**	4.72 ^b^ ± 0.24	5.91 ^a^ ± 1.03	6.20 ^a^ ± 0.83	6.94 ^a^ ± 0.03	3.66 ^a^ ± 0.00	3.68 ^a^ ± 0.09	3.90 ^a^ ± 0.10	4.17 ^a^ ± 0.01
**LnOP**	2.76 ^a^ ± 0.03	2.84 ^a^ ± 0.04	3.09 ^a^ ± 0.15	3.38 ^a^ ± 0.18	2.24 ^a^ ± 0.03	2.52 ^a^ ± 0.23	2.90 ^a^ ± 0.17	3.38 ^a^ ± 0.28
**OLO**	0.32 ^a^ ± 0.03	0.34 ^a^ ± 0.04	0.31 ^a^ ± 0.01	0.37 ^a^ ± 0.00	0.28 ^a^ ± 0.01	0.30 ^a^ ± 0.00	0.40 ^a^ ± 0.04	0.41 ^a^ ± 0.07
**SOLn**	1.61 ^b^ ± 0.05	1.99 ^a^ ± 0.39	1.93 ^b^ ± 0.23	2.20 ^a^ ± 0.01	1.22 ^a^ ± 0.01	1.38 ^a^ ± 0.01	1.64 ^b^ ± 0.04	1.91 ^a^ ± 0.03
**OOO**	0.41 ^b^ ± 0.08	0.57 ^a^ ± 0.21	0.81 ^b^ ± 0.24	1.11 ^a^ ± 0.20	0.20 ^a^ ± 0.01	0.23 ^a^ ± 0.02	0.25 ^b^ ± 0.04	0.44 ^a^ ± 0.03
**SOO**	0.16 ^a^ ± 0.06	0.19 ^a^ ± 0.07	0.22 ^a^ ± 0.07	0.25 ^a^ ± 0.01	—	—	—	—

Where: U = Unroasted, R = Roasted; Mean ± standard deviation; Number of replicates for each analysis: 3; Values with different letters (^a^, ^b^) are significantly different (*p* < 0.05).

**Table 6:** Tocochromanol (mg/100g) and phytosterol composition (mg/g) of oil extracted from four Egyptian unroasted and roasted linseed varieties.

	Egyptian Linseed Cultivars							
Tocochromanols (mg/100 g)	Giza 11		Giza 12		Sakha 3		Sakha 6	
	U	R	U	R	U	R	U	R
**alpha-T**	0.47 ^a^ ± 0.01	0.45 ^a^ ± 0.01	0.46 ^a^ ± 0.02	0.37 ^b^ ± 0.01	0.47 ^a^ ± 0.01	0.37 ^b^ ± 0.02	0.45 ^a^ ± 0.01	0.31 ^b^ ± 0.01
**gamma-T**	51.28 ^a^ ± 0.04	51.42 ^a^ ± 0.05	55.69 ^a^ ± 0.22	51.71 ^b^ ± 0.09	49.39 ^a^ ± 0.04	49.72 ^a^ ± 0.04	45.27 ^a^ ± 0.02	40.53 ^b^ ± 0.11
**delta-T**	0.65 ^a^ ± 0.03	0.66 ^a^ ± 0.01	0.77 ^a^ ± 0.02	0.70 ^b^ ± 0.02	0.54 ^a^ ± 0.03	0.48 ^b^ ± 0.01	0.55 ^a^ ± 0.02	0.46 ^b^ ± 0.01
**sum-T**	52.40 ^a^ ± 0.01	52.53 ^a^ ± 0.03	56.92 ^a^ ± 0.22	52.78 ^b^ ± 0.09	50.39 ^a^ ± 0.05	50.56 ^a^ ± 0.06	46.27 ^a^ ± 0.05	41.29 ^b^ ± 0.11
**Plastochromanol-8**	15.93 ^b^ ± 0.08	18.08 ^a^ ± 1.59	15.26 ^b^ ± 0.07	19.15 ^a^ ± 0.14	15.77 ^b^ ± 0.13	23.13 ^a^ ± 0.12	14.74 ^b^ ± 0.16	17.62 ^a^ ± 0.03
**Phytosterols (mg/g)**								
**Campesterol**	0.93 ^a^ ± 0.08	0.85 ^a^ ± 0.04	0.80 ^a^ ± 0.03	0.77 ^a^ ± 0.08	0.77 ^b^ ± 0.05	0.90 ^a^ ± 0.01	0.74 ^a^ ± 0.02	0.73 ^a^ ± 0.01
**Campestanol**	0.14 ^a^ ± 0.01	0.13 ^a^ ± 0.00	0.12 ^a^ ± 0.00	0.10 ^a^ ± 0.01	0.11 ^a^ ± 0.01	0.12 ^a^ ± 0.01	0.13 ^a^ ± 0.02	0.12 ^a^ ± 0.02
**Stigmasterol**	0.29 ^a^ ± 0.02	0.27 ^a^ ± 0.01	0.27 ^a^ ± 0.01	0.29 ^a^ ± 0.01	0.24 ^a^ ± 0.02	0.27 ^a^ ± 0.00	0.20 ^a^ ± 0.00	0.19 ^a^ ± 0.01
**β-Sitosterol**	1.54 ^a^ ± 0.10	1.39 ^b^ ± 0.04	1.39 ^a^ ± 0.09	1.31 ^a^ ± 0.13	1.25 ^b^ ± 0.07	1.45 ^a^ ± 0.02	1.32 ^a^ ± 0.04	1.33 ^a^ ± 0.04
**Sitostanol**	0.19 ^a^ ± 0.01	0.15 ^a^ ± 0.03	0.15 ^a^ ± 0.02	0.17 ^a^ ± 0.03	0.18 ^a^ ± 0.02	0.19 ^a^ ± 0.02	0.22 ^a^ ± 0.04	0.15 ^b^ ± 0.01
**Δ5-Avenasterol**	0.52 ^a^ ± 0.03	0.43 ^b^ ± 0.02	0.40 ^a^ ± 0.04	0.38 ^a^ ± 0.05	0.46 ^a^ ± 0.03	0.51 ^a^ ± 0.00	0.50 ^a^ ± 0.00	0.46 ^a^ ± 0.01
**Δ7-Stigmasterol**	1.44 ^a^ ± 0.08	1.33 ^a^ ± 0.06	1.46 ^a^ ± 0.08	1.41 ^a^ ± 0.10	1.32 ^a^ ± 0.05	1.38 ^a^ ± 0.00	1.39 ^a^ ± 0.01	1.32 ^a^ ± 0.02
**Cycloartenol**	0.31 ^a^ ± 0.01	0.28 ^a^ ± 0.01	0.34 ^a^ ± 0.03	0.32 ^a^ ± 0.02	0.25 ^a^ ± 0.01	0.25 ^a^ ± 0.00	0.30 ^a^ ± 0.00	0.28 ^a^ ± 0.01
**24-Methylenecycloartanol**	0.05 ^a^ ± 0.00	0.04 ^a^ ± 0.01	0.05 ^a^ ± 0.00	0.06 ^a^ ± 0.01	0.05 ^a^ ± 0.01	0.04 ^a^ ± 0.00	0.04 ^a^ ± 0.01	0.06 ^a^ ± 0.01

Where: U = Unroasted, R = Roasted; Mean ± standard deviation; Number of replicates for each analysis: 3; Values with different letters (^a^, ^b^) are significantly different (*p* < 0.05).

**Table 7:** EC 50 mg/mL (calculated from DPPH scavenging activity) and radical scavenging activity (RSA%) of linseed oil extracted from four unroasted and roasted Egyptian linseed cultivars.

	Egyptian Cultivars							
Parameters	Giza 11		Giza 12		Sakha 3		Sakha 6	
	U	R	U	R	U	R	U	R
**RSA%**	17.14 ^a^ ± 1.46	17.5 1^a^ ± 0.91	15.73 ^b^ ± 0.32	21.04 ^a^ ± 0.75	20.22 ^a^ ± 0.37	19.64 ^a^ ± 0.86	18.40 ^a^ ± 0.51	17.63 ^a^ ± 0.39
* EC_50_ mg/mL	26.84 ^a^	24.52 ^b^	25.9 7^a^	23.47 ^b^	24.67 ^a^	23.01 ^a^	29.12 ^a^	26.18 ^b^

Where: U = Unroasted, R = Roasted; * Equivalent to 0.04 mg/ml EC50 for Trolox; Mean ± standard deviation; Number of replicates for each analysis: 3; Values with different letters (^a^, ^b^) are significantly different (*p* < 0.05).

## Data Availability

The data presented in this study are available on request from the corresponding author.
